# Oxidation and reduction data of four subphthalocyanines with axially coordinated ferrocenylcarboxylic acids

**DOI:** 10.1016/j.dib.2020.105816

**Published:** 2020-06-03

**Authors:** Pieter J. Swarts, Jeanet Conradie

**Affiliations:** Department of Chemistry, PO Box 339, University of the Free State, Bloemfontein, 9300, South Africa

**Keywords:** Ferrocenylsubphthalocyanine, Cyclic voltammetry, Oxidation, Electronic effect, Ferrocenylcarboxylic acid

## Abstract

Redox data obtained from cyclic voltammetry experiments of the Fe^II/III^ and ring-based oxidation and reductions of subphthalocyanines containing a ferrocenylcarboxylic acid as axial ligand, is presented in this data in brief article. The Fe^II/III^ oxidation of ferrocenylsubphthalocyanines which containing the electron-withdrawing fluorine atoms at the peripheral and non-peripheral positions, are *ca.* 0.100 V more positive than Fe^II/III^ oxidation of ferrocenylsubphthalocyanines containing hydrogens at the peripheral and non-peripheral positions. For more insight into the reported data, see the related research article “*Redox and photophysical properties of four subphthalocyanines containing ferrocenylcarboxylic acid as axial ligands*” [1].

**Specifications Table**

**Table:** 

Subject	Chemistry
Specific subject area	Electrochemistry
Type of data	Table Image Graph Figure
How data were acquired	Princeton Applied Research PARSTAT 2273 potentiostat running Powersuite software (Version 2.58).
Data format	Raw Analysed
Parameters for data collection	Samples were used as synthesized. All the electrochemical experiments were performed in an M Braun Lab Master SP glove box, under a high purity argon atmosphere (H_2_O and O_2_ < 10 ppm).
Description of data collection	All electrochemical experiments were conducted in a 2 ml electrochemical cell containing three-electrodes (a glassy carbon working electrode, a Pt auxiliary electrode and a Pt pseudo reference electrode), connected to a Princeton Applied Research PARSTAT 2273 electrochemical analyser. Data obtained was exported to excel for analysis and diagram preparation.
Data source location	Institution: University of the Free State City/Town/Region: Bloemfontein Country: South Africa
Data accessibility	With the article
Related research article	P.J. Swarts, J. Conradie, Redox and photophysical properties of four subphthalocyanines containing ferrocenylcarboxylic acid as axial ligands [1].

**Value of the Data**

• The electrochemistry of subphthalocyanines provides insight and understanding into the macrocyclic ring-based oxidation and reduction processes. Introducing a ferrocenyl unit at the axial position of a subphthalocyanine, has a strong influence on the optical and redox properties of the ferrocenylsubphthalocyanines. Several ferrocenylsubphthalocyanines showed photo-induced electron-transfer properties that are important for solar devices which convert sunlight into electricity. Different axial ligands and ring substituents can fine-tune the redox properties of subphthalocyanines for use in different applications. This data provides detailed redox data of four ferrocenylsubphthalocyanines containing different axial ligands and different ring substituents.
• The data reported here provides insight for electrochemists into the effect of both electron-rich or electron-poor macrocycles of ferrocenylsubphthalocyanines Y-BSubPc(H)_12_ and Y-BSubPc(F)_12_ respectively, on the iron(II/III) oxidation potential of the ferrocenylcarboxylic acid ligand Y in the axial position. Axial ligand Y = either a non π-communicating (Fc**-CH_2_-CH_2_-**COO-) or a π-communicating (Fc**-CH=CH-**COO-) ferrocenyl moiety.
• Availability of electrochemical data of both the iron(II/III) and ring-based oxidation and reduction processes, assisting in future research in designing ferrocenylsubphthalocyanines with specific redox properties.

## 1 Data Description

**Figure 1:**
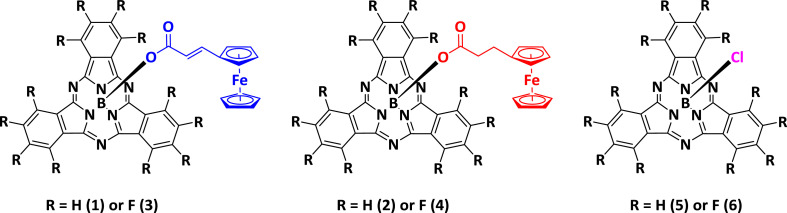
Structure of compounds in this study: (Fc(CH)_2_COO)-BSubPc(H)_12_, **1**, (Fc(CH_2_)_2_COO)-BSubPc(H)_12_, **2**, (Fc(CH)_2_COO)-BSubPc(F)_12_, **3**, (Fc(CH_2_)_2_COO)-BSubPc(F)_12_, **4**, Cl-BSubPc(H)_12_, **5**, and Cl-BSubPc(F)_12_, **6**.

**Figure 2:**
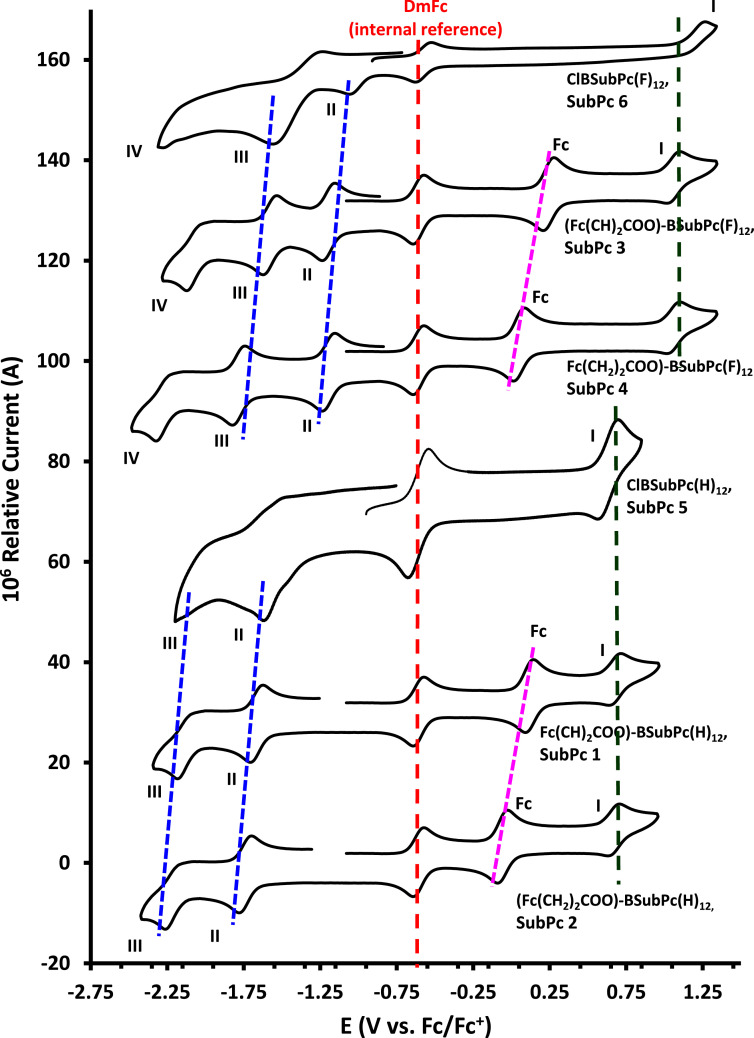
Cyclic voltammograms in DCM, at scan rate 0.200 Vs^−1^, of compounds **1** – **6**: (Fc(CH)_2_COO)-BSubPc(H)_12_, **1**, (Fc(CH_2_)_2_COO)-BSubPc(H)_12_, **2**, (Fc(CH)_2_COO)-BSubPc(F)_12_, **3**, (Fc(CH_2_)_2_COO)-BSubPc(F)_12_, **4**, Cl-BSubPc(H)_12_, **5** and Cl-BSubPc(F)_12_, **6**. CV's of **5** and **6** were obtained from [2]. Top three scans show the fluorine-substituted compounds (**3, 4, 6**), while bottom three scans contain no fluorine (**1, 2, 5**). Scans were initiated in a positive direction from *ca.* -1 V. Concentration of compounds **1** – **6** = 0.0005 mol dm^−3^ and of supporting electrolyte [N(*^n^*Bu)_4_][B(C_6_F_5_)_4_] = 0.1 mol dm^−3^.

**Figure 3:**
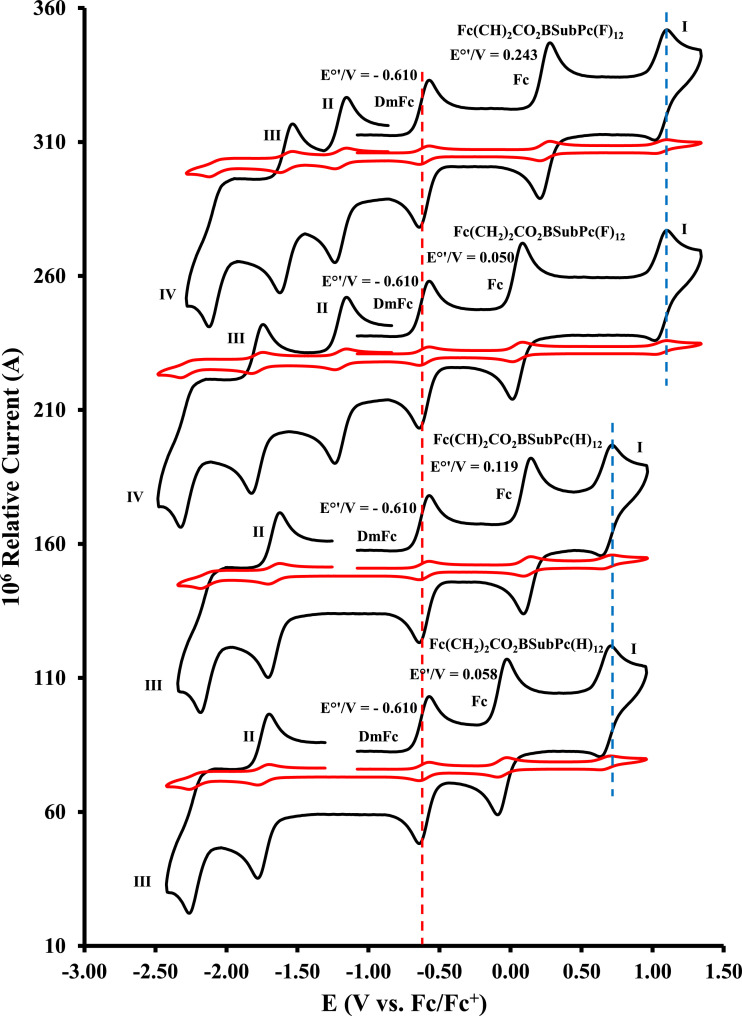
Cyclic voltammograms in DCM, at scan rates 0.050 Vs^−1^ (red) and 5.00 Vs^−1^ (black) for compounds **1** – **4**, from bottom to top: Fc(CH_2_)_2_CO_2_-BSubPc(H)_12_, **2**, Fc(CH)_2_CO_2_-BSubPc(H)_12_, **1**, Fc(CH_2_)_2_CO_2_-BSubPc(F)_12_, **4**, and Fc(CH)_2_CO_2_-BSubPc(F)_12_, **3**. Scans were initiated in a positive direction from *ca.* -1 V. Data for the formal reduction potential (E^0’^) of the internal standard DmFc (left peak, red dotted line), and of ferrocene oxidation of the axial ligand (marked as Fc), are indicated in V. Concentration of compounds **1** – **4** = 5 × 10^−4^ mol dm^−3^ and of supporting electrolyte [N(*^n^*Bu)_4_][B(C_6_F_5_)_4_] = 0.1 mol dm^−3^.

**Figure 4:**
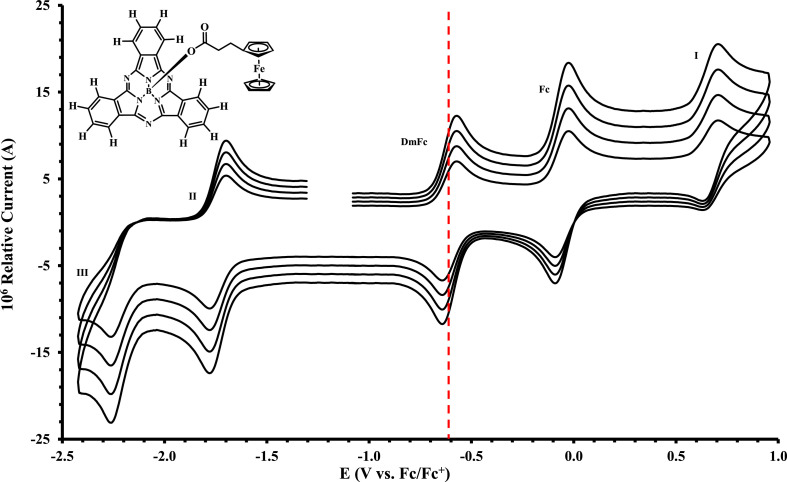
Cyclic voltammograms in DCM of Fc(CH_2_)_2_CO_2_-BSubPc(H)_12_ (compound **2**), at scan rates 0.200 (smallest peak current), 0.300, 0.400 and 0.500 Vs^−1^ (largest peak current). Scans were initiated in a positive direction from *ca.* -1 V, with the DmFc internal standard peak at the red dotted line. Concentration of analyte = 5 × 10^−4^ mol dm^−3^ and of supporting electrolyte [N(*^n^*Bu)_4_][B(C_6_F_5_)_4_] = 0.1 mol dm^−3^.

**Figure 5:**
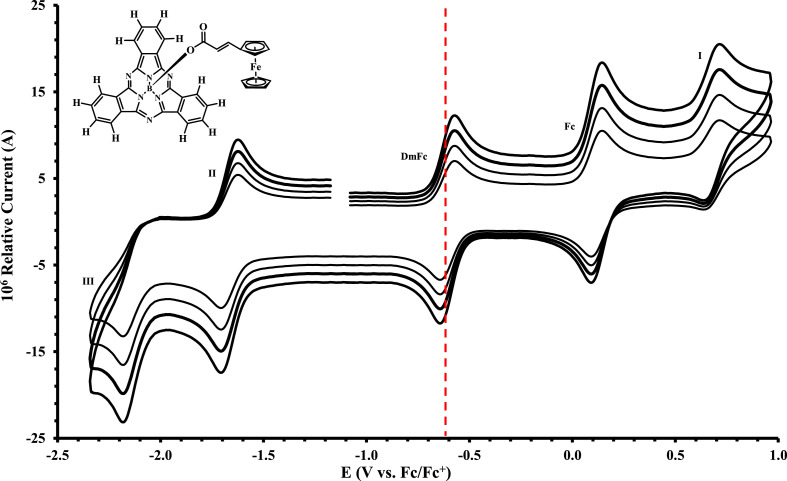
Cyclic voltammograms in DCM of Fc(CH)_2_CO_2_-BSubPc(H)_12_ (compound **1**), at scan rates 0.200 (smallest peak current), 0.300, 0.400 and 0.500 Vs^−1^ (largest peak current). Scans were initiated in a positive direction from *ca.* -1 V, with the DmFc internal standard peak at the red dotted line. Concentration of analyte = 5 × 10^−4^ mol dm^−3^ and of supporting electrolyte [N(*^n^*Bu)_4_][B(C_6_F_5_)_4_] = 0.1 mol dm^−3^.

**Figure 6:**
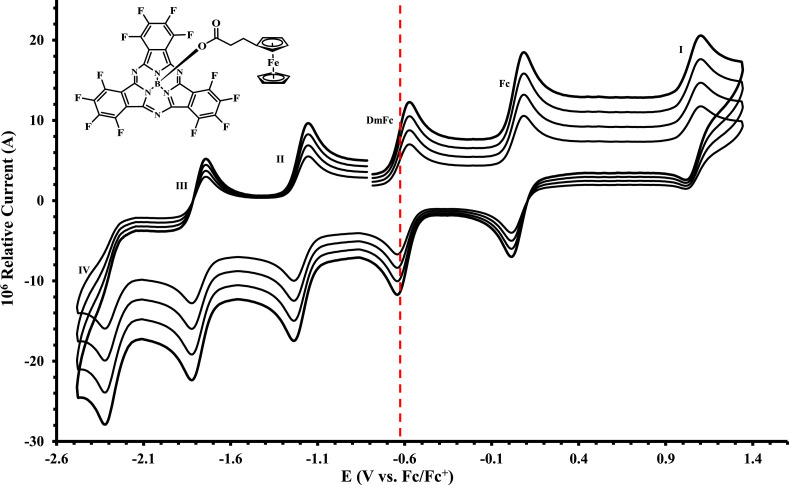
Cyclic voltammograms in DCM of Fc(CH_2_)_2_CO_2_-BSubPc(F)_12_ (compound **4**), at scan rates 0.200 (smallest peak current), 0.300, 0.400 and 0.500 Vs^−1^ (largest peak current). Scans were initiated in a positive direction from *ca.* -1 V, with the DmFc internal standard peak at the red dotted line. Concentration of analyte = 5 × 10^−4^ mol dm^−3^ and of supporting electrolyte [N(*^n^*Bu)_4_][B(C_6_F_5_)_4_] = 0.1 mol dm^−3^.

**Figure 7:**
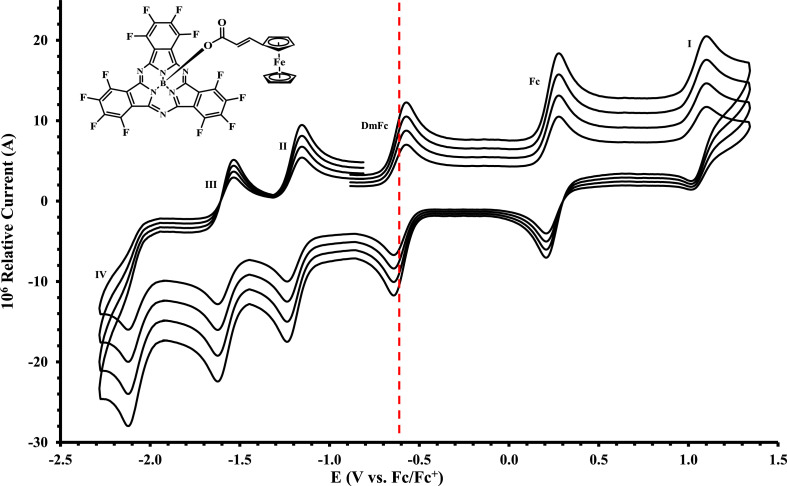
Cyclic voltammograms in DCM of Fc(CH)_2_CO_2_-BSubPc(F)_12_ (compound **3**), at scan rates 0.200 (smallest peak current), 0.300, 0.400 and 0.500 Vs^−1^ (largest peak current). Scans were initiated in a positive direction from *ca.* -1 V, with the DmFc internal standard peak at the red dotted line. Concentration of analyte = 5 × 10^−4^ mol dm^−3^ and of supporting electrolyte [N(*^n^*Bu)_4_][B(C_6_F_5_)_4_] = 0.1 mol dm^−3^.

The electrochemical data of ferrocenylsubphthalocyanines **1** – **4** shown in Figure 1 is summarized in Table 1, Table 2, Table 3, Table 4, with the cyclic voltammograms (CVs) shown in Figure 2, Figure 3, Figure 4, Figure 5, Figure 6, Figure 7. Raw cyclic voltammetric data is available in excel format as supplementary data files. Comparative CVs, comparing the shift in the CV data of these ferrocenylsubphthalocyanines relative to the known chloro-subphthalocyanines (**5** and **6**) [2], are shown in Figure 2. The ferrocenylcarboxylic acid axial ligand causes the reduction peaks of the ferrocenylsubphthalocyanines to shift more negative relative to the chlorosubphthalocyanines. Cyclic voltammograms of the fluorinated subphthalocyanines **3** and **4**, showed one iron-based and one ring-based oxidation as well as three ring-based reductions. Cyclic voltammograms of the non-fluorinated subphthalocyanines **1** and **2**, also showed one iron-based and one ring-based oxidation, but only two ring-based reductions. Previous studies showed that the first oxidation in related ferrocenylsubphthalocyanines is iron based [1,[3], [4], [5]]. The iron-based first oxidation in compounds **1** – **4** occurs at a lower potential than the first ring-based oxidation in **1** – **6**. Porphyrins [6], phthalocyanines [7] and subphthalocyanines (SubPcs) [8,9] can show up to three ring-based oxidations and three ring-based reductions. In most cases the first ring-based oxidation of the SubPcs exhibits irreversible behaviour [9]; however, in this case chemically reversible first ring-based oxidation, with peak current ratios of 1 and peak current separations of Δ*E* = 0.074 – 0.084 V, were obtained.

**Table 1:** Electrochemical data (potential in V *vs.* Fc/Fc^+^) in DCM for *ca*. 5 × 10^−4^ mol dm^−3^ of Fc(CH_2_)_2_CO_2_-BSubPc(H)_12_ (compound **2**), at indicated scan rates (ν in V/s). See Figure 4 for assigment of peaks.

**ν (V/s)**	***E***_**p^a^**_**/ V**	**Δ*****E***_**p**_**/ V**	***E***^**o**^**′ / V**	***i***_**p^b^**_**/ μA**	***i***_**p**_**/*****i***_**p'^c^**_
c**Fc**					
0.050	-0.021	0.073	-0.058	2.61	0.99
0.200	-0.020	0.075	-0.058	5.12	0.99
0.300	-0.020	0.076	-0.058	5.86	0.99
0.400	-0.019	0.077	-0.058	8.42	0.99
0.500	-0.019	0.078	-0.058	9.15	0.99
5.000	-0.018	0.080	-0.058	24.98	0.99
**Wave I**					
0.050	0.711	0.083	0.670	2.36	0.99
0.200	0.712	0.085	0.670	4.63	0.99
0.300	0.713	0.086	0.670	5.30	0.99
0.400	0.713	0.087	0.670	7.61	0.99
0.500	0.714	0.088	0.670	8.28	0.99
5.000	0.715	0.090	0.670	17.09	0.99
**Wave II**					
0.050	-1.782	0.083	-1.741	2.44	0.99
0.200	-1.783	0.085	-1.741	4.79	0.99
0.300	-1.784	0.086	-1.741	5.47	0.99
0.400	-1.784	0.087	-1.741	7.87	0.99
0.500	-1.785	0.088	-1.741	8.55	0.99
5.000	-1.786	0.090	-1.741	23.82	0.99
**Wave III**					
0.050	-2.263	**-**	**-**	**2.62**	**-**
0.200	-2.264	**-**	**-**	**5.82**	**-**
0.300	-2.265	**-**	**-**	**8.09**	**-**
0.400	-2.265	**-**	**-**	**9.05**	**-**
0.500	-2.266	**-**	**-**	**10.24**	**-**
5.000	-2.267	**-**	**-**	**25.40**	**-**

^a^*E_p_* is the peak anodic potential for oxidation (*E_ox_*) and peak cathodic potential for reduction (*E_red_*).

^b^*i_p_* is the peak anodic current for oxidation (*i_pa_*) and peak cathodic current for reduction (*i_pc_*).

^c^ peak current ratio = *i_pc_/i_pa_* for oxidation and *i_pa_/i_pc_* for reduction.

**Table 2:** Electrochemical data (potential in V *vs.* Fc/Fc^+^) in DCM for *ca*. 5 × 10^−4^ mol dm^−3^ of Fc(CH)_2_CO_2_-BSubPc(H)_12_ (compound **1**), at indicated scan rates (ν in V/s). See Figure 5 for assigment of peaks.

**ν (V/s)**	***E***_**p^a^**_**/ V**	**Δ*****E***_**p**_**/ V**	***E***^**o**^**′ / V**	***i***_**p^b^**_**/ μA**	***i***_**p**_**/*****i***_**p'^c^**_
**Fc**					
0.050	0.156	0.073	0.119	2.53	0.99
0.200	0.156	0.075	0.119	4.96	0.99
0.300	0.157	0.076	0.119	5.66	0.99
0.400	0.157	0.077	0.119	8.14	0.99
0.500	0.158	0.078	0.119	8.85	0.99
5.000	0.159	0.079	0.119	23.78	0.99
**Wave I**					
0.050	0.710	0.081	0.670	2.31	0.99
0.200	0.711	0.083	0.670	4.54	0.99
0.300	0.712	0.084	0.670	5.18	0.99
0.400	0.712	0.085	0.670	7.45	0.99
0.500	0.713	0.086	0.670	8.10	0.99
5.000	0.714	0.088	0.670	16.53	0.99
**Wave II**					
0.050	-1.703	0.083	-1.662	2.48	0.99
0.200	-1.704	0.085	-1.662	4.86	0.99
0.300	-1.705	0.086	-1.662	5.55	0.99
0.400	-1.705	0.087	-1.662	7.98	0.99
0.500	-1.706	0.088	-1.662	8.68	0.99
5.000	-1.707	0.090	-1.662	23.77	0.99
**Wave III**					
0.050	-2.183	**-**	**-**	**2.36**	**-**
0.200	-2.184	**-**	**-**	**5.24**	**-**
0.300	-2.185	**-**	**-**	**7.29**	**-**
0.400	-2.185	**-**	**-**	**8.62**	**-**
0.500	-2.186	**-**	**-**	**9.36**	**-**
5.000	-2.187	**-**	**-**	**24.57**	**-**

^a^*E_p_* is the peak anodic potential for oxidation (*E_ox_*) and peak cathodic potential for reduction (*E_red_*).

^b^*i_p_* is the peak anodic current for oxidation (*i_pa_*) and peak cathodic current for reduction (*i_pc_*).

^c^ peak current ratio = *i_pc_/i_pa_* for oxidation and *i_pa_/i_pc_* for reduction.

**Table 3:** Electrochemical data (potential in V *vs* Fc/Fc^+^) in DCM for *ca*. 5 × 10^−4^ mol dm^−3^ of Fc(CH_2_)_2_CO_2_-BSubPc(F)_12_ (compound **4**), at indicated scan rates (ν in V/s). See Figure 6 for assigment of peaks.

**ν (V/s)**	***E***_**p^a^**_**/ V**	**Δ*****E***_**p**_**/ V**	***E***^**o**^**′ / V**	***i***_**p^b^**_**/ μA**	***i***_**p**_**/*****i***_**p'^c^**_
**Fc**					
0.050	0.089	0.077	0.050	2.65	0.99
0.200	0.089	0.079	0.050	5.19	0.99
0.300	0.090	0.080	0.050	5.94	0.99
0.400	0.090	0.081	0.050	8.53	0.99
0.500	0.091	0.082	0.050	9.28	0.99
5.000	0.092	0.084	0.050	23.91	0.99
**Wave I**					
0.050	1.105	0.081	1.065	2.44	0.99
0.200	1.106	0.083	1.065	4.77	0.99
0.300	1.107	0.084	1.065	5.46	0.99
0.400	1.107	0.085	1.065	7.84	0.99
0.500	1.108	0.086	1.065	8.53	0.99
5.000	1.109	0.088	1.065	16.89	0.99
**Wave II**					
0.050	-1.239	0.085	-1.197	2.56	0.99
0.200	-1.240	0.087	-1.197	5.01	0.99
0.300	-1.241	0.088	-1.197	5.73	0.99
0.400	-1.241	0.089	-1.197	8.23	0.99
0.500	-1.242	0.090	-1.197	8.95	0.99
5.000	-1.243	0.092	-1.197	23.87	0.99
**Wave III**					
0.050	-1.824	0.087	-1.781	2.86	0.99
0.200	-1.825	0.089	-1.781	5.61	0.99
0.300	-1.826	0.090	-1.781	6.42	0.99
0.400	-1.826	0.091	-1.781	9.22	0.99
0.500	-1.827	0.092	-1.781	10.03	0.99
5.000	-1.828	0.094	-1.781	21.98	0.99
**Wave IV**					
0.050	-2.322	**-**	**-**	**2.46**	**-**
0.200	-2.323	**-**	**-**	**5.47**	**-**
0.300	-2.324	**-**	**-**	**7.61**	**-**
0.400	-2.324	**-**	**-**	**9.04**	**-**
0.500	-2.325	**-**	**-**	**9.89**	**-**
5.000	-2.326	**-**	**-**	**2.69**	**-**

^a^*E_p_* is the peak anodic potential for oxidation (*E_ox_*) and peak cathodic potential for reduction (*E_red_*).

^b^*i_p_* is the peak anodic current for oxidation (*i_pa_*) and peak cathodic current for reduction (*i_pc_*).

^c^ peak current ratio = *i_pc_/i_pa_* for oxidation and *i_pa_/i_pc_* for reduction.

**Table 4:** Electrochemical data (potential in V *vs.* Fc/Fc^+^) in DCM for *ca*. 5 × 10^−4^ mol dm^−3^ of Fc(CH)_2_CO_2_-BSubPc(H)_12_ (compound **3**), at indicated scan rates (ν in V/s). See Figure 7 for assigment of peaks.

**ν (V/s)**	***E***_**p^a^**_**/ V**	**Δ*****E***_**p**_**/ V**	***E***^**o**^**′ / V**	***i***_**p^b^**_**/ μA**	***i***_**p**_**/*****i***_**p'^c^**_
**Fc**					
0.050	0.282	0.077	0.243	2.60	0.99
0.200	0.282	0.079	0.243	5.10	0.99
0.300	0.283	0.080	0.243	5.82	0.99
0.400	0.283	0.081	0.243	8.37	0.99
0.500	0.284	0.082	0.243	9.10	0.99
5.000	0.285	0.084	0.243	24.38	0.99
**Wave I**					
0.050	1.106	0.083	1.065	2.41	0.99
0.200	1.107	0.085	1.065	4.72	0.99
0.300	1.108	0.086	1.065	5.39	0.99
0.400	1.108	0.087	1.065	7.75	0.99
0.500	1.109	0.088	1.065	8.43	0.99
5.000	1.110	0.090	1.065	17.22	0.99
**Wave II**					
0.050	-1.238	0.087	-1.195	2.47	0.99
0.200	-1.239	0.089	-1.195	4.84	0.99
0.300	-1.240	0.090	-1.195	5.54	0.99
0.400	-1.240	0.091	-1.195	7.96	0.99
0.500	-1.241	0.092	-1.195	8.65	0.99
5.000	-1.242	0.094	-1.195	22.59	0.99
**Wave III**					
0.050	-1.626	0.089	-1.582	2.78	0.99
0.200	-1.627	0.091	-1.582	5.45	0.99
0.300	-1.628	0.092	-1.582	6.22	0.99
0.400	-1.628	0.093	-1.582	8.95	0.99
0.500	-1.629	0.094	-1.582	9.73	0.99
5.000	-1.630	0.096	-1.582	20.94	0.99
**Wave IV**					
0.050	-2.126	**-**	**-**	**2.51**	**-**
0.200	-2.127	**-**	**-**	**5.59**	**-**
0.300	-2.128	**-**	**-**	**7.77**	**-**
0.400	-2.128	**-**	**-**	**9.19**	**-**
0.500	-2.129	**-**	**-**	**-10.02**	**-**
5.000	-2.130	**-**	**-**	**23.27**	**-**

^a^*E_p_* is the peak anodic potential for oxidation (*E_ox_*) and peak cathodic potential for reduction (*E_red_*).

^b^*i_p_* is the peak anodic current for oxidation (*i_pa_*) and peak cathodic current for reduction (*i_pc_*).

^c^ peak current ratio = *i_pc_/i_pa_* for oxidation and *i_pa_/i_pc_* for reduction.

## 2 Experimental Design, Materials and Methods

Electrochemical studies by means of cyclic voltammetric (CV) experiments were performed in an MBraun Lab Master SP glove box under a high purity argon atmosphere (H_2_O and O_2_ < 10 ppm), utilizing a Princeton Applied Research PARSTAT 2273 potentiostat, running Powersuite software (Version 2.58).

The cyclic voltammetry experimental setup consists of a cell with three electrodes, namely (i) a glassy carbon electrode as working electrode, (ii) a platinum wire auxiliary electrode and (ii) a platinum wire as pseudo reference electrode. The glassy carbon working electrode was polished and prepared before every experiment on a Buhler polishing mat, first with 1-micron and then with ¼-micron diamond paste, rinsed with H_2_O, acetone and DCM, and dried before each experiment.

Electrochemical analysis in dichloromethane as solvent (DCM, anhydrous, ≥ 99.8%, contains 40-150 ppm amylene as stabilizer) was conducted at RT. Solutions were made in 0.001 dm^3^ spectrochemical grade anhydrous DCM, containing *ca.* 0.0005 M of analyte, 0.0005 mol dm^−3^ of internal reference (decamethylferrocene, DmFc) and 0.1 mol dm^−3^ of supporting electrolyte tetrabutylammonium tetrakispentafluorophenylborate, [N(*^n^*Bu)_4_][B(C_6_F_5_)_4_] in DCM.

Experimental potential data was collected *vs.* the Pt wire reference electrode, measured *vs.* the redox couple of decamethylferrocene, DmFc, as internal standard and reported *vs.* the redox couple of ferrocene, Fc, as suggested by IUPAC [10]. E°′(DmFc) = - 0.610 V *vs.* Fc/Fc^+^ at 0 V in DCM/[N(*^n^*Bu)_4_][B(C_6_F_5_)_4_]. Scan rates were between 0.05 and 5.00 Vs^−1^.

## Declaration of Competing Interest

The authors declare that they have no known competing financial interests or personal relationships which have, or could be perceived to have, influenced the work reported in this article.

## Appendix Supplementary materials

Image, application 1

Image, application 2

Image, application 3

Image, application 4

Image, application 5

Image, application 6

Supplementary files: Raw data of all cyclic voltammograms are supplied in Excel files. Image, application 7
